# Continuous Flash Glucose Monitoring in children with Congenital Hyperinsulinism; first report on accuracy and patient experience

**DOI:** 10.1186/s13633-018-0057-2

**Published:** 2018-03-27

**Authors:** Hussain Alsaffar, Lucy Turner, Zoe Yung, Mohammed Didi, Senthil Senniappan

**Affiliations:** 1 Department of Paediatrics, Leighton Hospital, Crewe, CW1 4QJ UK; 2 Department of Paediatric Endocrinology, Alder Hey Children’s Hospital, L14 5AB, Liverpool, UK

**Keywords:** Hyperinsulinism, Flash glucose monitoring, FreeStyle libre

## Abstract

**Background:**

The factory calibrated FreeStyle Libre (FSL) flash glucose monitoring system has been recently introduced for use in patients with diabetes mellitus. There are no reports available regarding its use in patients with congenital hyperinsulinism (CHI). We have assessed the accuracy of FSL compared to the finger prick capillary blood glucose (CBG) over 2 weeks period in patients with CHI and evaluated the parents’ experience of using FSL.

**Methods:**

Four hundred sixty-seven episodes of CBG along with corresponding swipe FSL readings were available from 11 children with CHI (0.5–5 years). A detailed questionnaire was completed by the parents.

**Results:**

The mean variation between the two methods was 0.29 mmol/l (SD ±1.07), higher readings by FSL compared to CBG. The FSL sensors stayed in-situ for an average period of 11.5 days. There was a positive correlation between the two methods (*r* = 0.7). The FSL tended to overestimate compared to CBG (bias = 0.29 mmol/l; 95% CI: 0.19 to 0.38). Only 70% of values were within the reference standard (±0.83 mmol/l) at glucose concentrations less than 5.6 mmol/l. The overall Mean Absolute Relative Difference (MARD) was 17.9%. Forty two episodes of hypoglycaemia (CBG < 3.5 mmol/l) were noted but FSL identified only 52% of these episodes. The Bland Altman analysis showed the 95% limits of agreement between the two methods ranging from − 1.8 (95% CI: -1.97 to − 1.64) to 2.37 (95% CI: 2.21 to 2.54). Majority of the parents found the glucose trend on FSL to be useful to detect and prevent hypoglycaemic episodes. All parents felt that FSL is a very easy and convenient method to measure the glucose especially during sleep. A significant proportion of parents felt that FSL readings were not accurate and 56% of parents expressed interest to continue using FSL after the trial period.

**Conclusion:**

Noticeable variability between the two methods of measuring the glucose was noted. Despite the ease of using the FSL system, concerns related to accuracy, especially at low glucose values do remain although parents find the glucose trend to be very useful. Further larger trials are needed in CHI patients before FSL is recommended as a routine alternative method for measuring glucose levels.

## Background

Congenital hyperinsulinism (CHI) is the most common cause of severe persistent hypoglycaemia in children, mainly during infancy [1]. As the hyperinsulinism inhibits ketogenesis, the brain is deprived of glucose as well ketone bodies, thereby increasing the risk of neurological damage [2]. Hence it is important to maintain normoglycaemia in these patients to avoid neurodevelopmental issues. Majority of the patients with CHI are either infants or preschool children and the blood glucose levels are monitored on a regular basis by the parents using the standard glucometers.

Continuous glucose monitoring systems (CGMS) are mainly used for patients with diabetes mellitus. Randomised controlled trials using CGMS in adults with diabetes mellitus had revealed lower incidence of hypoglycaemia [3] and improved glycaemic control [4]. Very little information is available about the usage of CGMS in CHI patients. In a report involving the use of CGMS in a single patient with CHI, it was shown that CGMS could help the glycaemic control by showing the trend of interstitial glucose values [5]. Conrad et al. considered it as a useful adjunct in the diagnosis and evaluation of hypoglycaemia, and for documentation of euglycaemia in five patients with hypoglycaemic disorders [6]. However, the use of CGMS is not widespread in the management of CHI patients for various reasons including the cost of the equipment, the lack of evidence, the need for regular calibration for accuracy [7] and the short lifespan of the sensors.

Recently, the factory calibrated FreeStyle Libre (FSL, Abbot Diabetes Care, Alameda, California, USA) flash glucose monitoring system (FGMS) has been introduced for use in patients with diabetes mellitus [8]. It continuously measures the interstitial glucose concentration via disposable electronics and a subcutaneous sensor, with a button-like structure firmly adhering to the skin to allow the inserted sensor to stay in place for 14 days compared to less than 7 days using a standard CGMS. The sensor is put in place by a single-use applicator, and automatically measures glucose every minute for up to 14 days. Scanning of the sensor by a separate reader collects the glucose measurements and trend at the moment of scanning plus up to 8 h of prior readings every 15 min. In principle, the glucose sensing technique is based on the technique of the FreeStyle Navigator, which has been shown to be a reliable CGM measurement technique.

The first study to evaluate performance and usability of FSL in children with type 1 diabetes mellitus has demonstrated good agreement between sensor and CBG and the overall mean absolute relative difference (MARD) was 13.9% [9]. To our knowledge, no study has been undertaken so far to evaluate the use of FSL in CHI patients.

In this study, we assessed the accuracy of FSL compared to the finger prick capillary blood glucose (CBG) over 2-week period in patients with CHI and assessed the parents’ experience of using FSL.

## Methods

Eleven patients [aged 0.5–5 years (7 M); median age 2 years], who had persistent CHI for more than 6 months (requiring diazoxide therapy) were recruited into this prospective study. The parents were advised to swipe the FSL for glucose readings every time they record the CBG (using Abbott Freestyle optimum neo blood glucose meter). Parents were advised to monitor CBG at least 4 times per day (pre-feed) and manage the hypoglycaemic episodes as per standard clinical care plan based on the CBG reading. As per standard care plan, a low blood glucose value (< 3.5 mmol/l) is rechecked within 10 min and acted upon if still low by administering glucose gel or feed. Each patient was given one sensor; therefore, the maximum duration of possible comparison was 14 days per patient, as the sensor should be replaced after this period. When the sensor was removed, parents were asked to fill a questionnaire that reflects their experience of using the FGMS for checking the blood glucose. Institutional approval was obtained and verbal consent was given by all the parents prior to participation in the study.

## Results

Four hundred sixty-seven episodes of CBG along with the corresponding swipe FSL readings (average of 4.3 swipes per day) were available from the 11 (7 M) patients with diffuse CHI aged (0.5–5 years), 6 of them were negative mutations for in *ABCC8, KCNJ11, GLUD1, GCK, HADH, HNF4A, INSR, HNF1A*.

### Accuracy of FGMS in CHI patients

The mean variation between the two methods was 0.29 mmol/l (SD ±1.07), higher readings by FSL compared to CBG. The FSL sensor stayed in-situ for an average period of 11.5 days (2-14 days). There was a positive correlation between the two methods (*r* = 0.7) with statistical significant relation (*p* < 0.05).

**Fig. 1:**
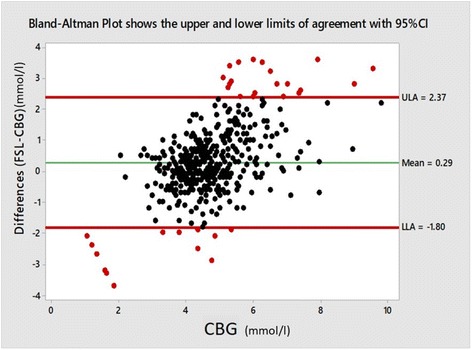
Bland-Altman plot shows the lower and upper limits of agreement with 95% CI. -1.8 (95% CI: -1.97 to − 1.64) to 2.37 (95% CI: 2.21 to 2.54)

The FSL tended to overestimate compared to CBG with a positive mean difference, (bias = 0.29 mmol/l; 95% CI: 0.19 to 0.38). Bland Altman analysis revealed the 95% limits of agreement between the two methods ranged from − 1.8 (95% CI: -1.97 to − 1.64) to 2.37 (95% CI: 2.21 to 2.54) (Fig. 1). The Passing Bablok regression showed intercept A of − 2.27 (95% CI -2.9 to − 1.6) and slope B of 1.57 (95% CI 1.42 to 1.72). These results lead to a conclusion that both methods differ at least by a constant amount and there is at least a proportional difference between them (Fig. 1).

**Fig. 2:**
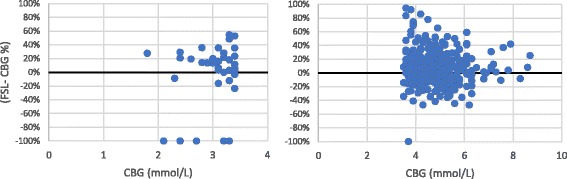
Difference plot between FSL and CBG when CBG < 3.5 mmol/l (left) and when CBG ≥3.5 mmol/l (right)

Seventy percent of FSL values were within the International Organization for Standardization (ISO) reference standard (±0.83 mmol/l) when capillary glucose concentrations were less than 5.6 mmol/l [10]. Forty-two episodes of symptomatic hypoglycaemia (CBG < 3.5 mmol/l) were noted. FSL identified 52% of these episodes. The difference plot between FSL and CBG illustrated overestimation in 66% of the cases by + 3% to + 55% of CBG readings during hypoglycaemia (Fig. 2(left)). Correlation coefficient was positive (*r* = 0.45), whereas the correlation coefficient was better when CBG was ≥3.5 mmol/l (*r* = 0.65). The overestimation of FSL readings was noted in 57% of readings when CBG ≥3.5 mmol/l. In 3.8% of cases, the relative difference between FSL and CBG was more than + 50% when CBG ≥3.5 mmol/l [Fig. 2(right)]. The overall Mean Absolute Relative Difference (MARD) was 17.9%.

**Fig. 3:**
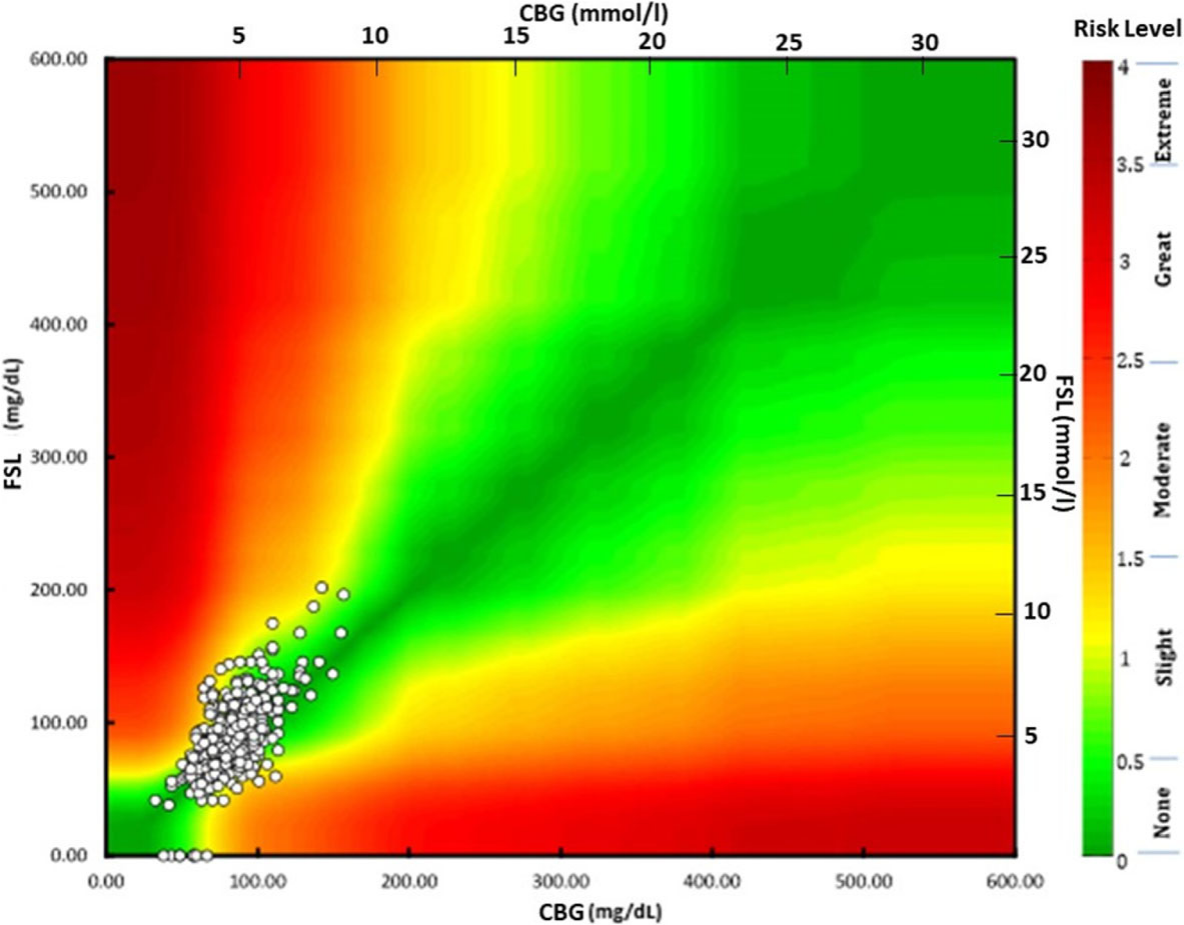
Wakeman colour surveillance error grid analysis demonstrates risk levels based on absolute difference between CBG and FSL glucose values

Wakeman’s colour-coded surveillance error grid highlighted none to moderate risk level with regards to absolute difference between the two methods in CHI patients (Fig. 3). This colour surveillance error grid analysis illustrates risk levels; none means no effect on clinical action as FSL is almost same as CBG, slight risk indicates altered clinical action, but little or no effect on the clinical outcome. Moderate risk means the difference is likely to affect the outcome, whereas great and extreme risk levels mean that the difference between FSL and CBG can lead to significant medical risk and dangerous consequences.

### Parental questionnaire results

Eleven questionnaires were completed and returned by the parents of the CHI patients. All of them agreed that FSL reader was easy to understand and the measuring of glucose using the FSL technology during activities was excellent. The majority found the glucose trend on FSL to be very useful to detect and prevent hypoglycaemic episodes. Four parents thought it had made positive impact on the quality of life of their child. Two other parents thought it has improved their own quality of life rather than that of the child. On a scale of (1-5) their responses varied (Table 1).

**Table 1:** Parental responses to the questionnaire completed at the end of FSL trial period

Areas addressed in the questionnaire	Parental response on 1-5 scale (1 = strongly disagree and 5 = strongly agree) Therefore response 1 or 2 were taken as negative and response 4 or 5 positive while response 3 meant equivocal response.		
	Yes (%)	No (%)	Equivocal (%)
Easy to attach the FSL sensor	81	0	19
Pain associated with the insertion of the sensor	45.3	27.3	27.3
Comfortable to use the FSL sensor	81.8	9.1	9.1
Visibility of the sensor	27.3	9.1	63.6
Accuracy of FSL	18.2	63.6	18.2
Confidence of parents to rely on FSL	36.6	45.3	18.2

Two parents felt that the FSL sensor insertion was extremely painful. 77% of the parents felt that FSL is not always reliable. 56% of parents would like to continue using FSL and half of this group would probably purchase the FSL sensors after the end of trial period. The majority felt that FSL is a very convenient method to measure the glucose level during sleep. It was reported that one sensor stopped working after bathing the 3 years old toddler and in another case; it was pulled out by the 18 months old just 2 days after the insertion of the sensor.

## Discussion

CHI is a condition that requires intense glucose monitoring especially at the time of establishing the underlying diagnosis and trialling the definitive therapy. Hyperinsulinism leads to switching off ketogenesis; hence no alternative fuel to brain is available in the hyperinsulinaemic hypoglycaemic state. The need to monitor the glucose carefully and regularly to avoid hypoglycaemia is a key aspect of the initial and subsequent management pathways [11]. Long term glucose monitoring is essential to detect and appropriately manage the hypoglycaemic episodes, as asymptomatic hypoglycaemic episodes are not uncommon in these children.

Over the last 15 years, manufacturers have used the advances in technology to produce different devices and systems to record and monitor the blood glucose. A practical, user-friendly, easy device to monitor the blood glucose is deemed necessary due to the frequency of its use. Over the last 10 years, there were two main categories of devices that were developed to monitor the blood glucose outside hospital settings; the conventional CBG and the CGMS.

Couple of years ago, Abbot brought to the market, the FreeStyle Libre (FSL) reader that harnesses the advanced sensor-based technology to read the interstitial glucose and to instantly display the data in a user-friendly way [12]. FSL is felt to be very convenient as the sensor measures and continuously saves glucose readings without the need to prick the skin frequently to check the glucose level. It was felt to be practical as the reader can capture the data from the sensor over the clothing. In addition, the advantages of the sensor include the fact that it is water resistant, of small size, designed to stay in the body for 14 days and requires no finger prick calibration [12]. It has been shown that FGM is accurate compared with capillary blood glucose reference values over 14 days in diabetes mellitus in both children and adults [7, 13]. However, it has been recommended to use finger prick test when the blood glucose levels are changing rapidly as per the manufacturer’s advice.

CHI is not as common as diabetes mellitus; hence there is paucity of trials and reports with regards to continuous glucose monitoring. Monsod et al. reported that CGMS could accurately track acute changes in plasma glucose when calibrated across a range of plasma glucose and insulin levels. However, hyperinsulinemia may contribute to modest discrepancies between plasma and sensor glucose levels [14]. The same was noted in our study, as MARD in this cohort of patients was higher than the MARD when FSL was studied in patients with type 1 diabetes mellitus. It was reported to be 10.9% in a small cohort of patients [15] and 13.9% in multicentre study that involved 89 participants with diabetes mellitus (4-17 years old) [16].

The correlation between the two methods was less positive during the hypoglycaemic events as FSL tended to overestimate the true glucose values. This has its adverse effects from practical point of view, as patients may have not been treated when they are truly hypoglycaemic. This has obviously generated anxiety and less confidence amongst the parents to rely on FSL method to detect and manage hypoglycaemia. The overall positive correlation, especially when CBG ≥ 3.5 mmol/l gave the parents an opportunity to act on the trend of the glucose as it helped them to reduce the incidence of hypoglycaemic episodes when the trend goes downward.

The inaccuracy of FGM in patients with CHI could have been related to the nature of the underlying condition and their young age with possible impact on muscle/fat ratio compared to older cohort of patients with diabetes mellitus. The limitations of the study include small sample size and short duration and hence, a larger prospective study is required to further elucidate the role of FGM in CHI.

## Conclusion

Noticeable variability between the two methods of measuring the glucose was noted. Despite the ease of using the FSL system, concerns related to accuracy, especially at low glucose values do remain although parents find the glucose trend to be very useful. Further larger trials are needed in CHI patients before FSL is recommended as a routine alternative method for measuring glucose values.

## Abbreviations

CBG: Capillary blood glucose
CGMS: Continuous glucose monitoring systems
CHI: Congenital hyperinsulinism
FSL: FreeStyle Libre
MARD: Mean Absolute Relative Difference
